# The Need for Orthodontic Treatment among Vietnamese School Children and Young Adults

**DOI:** 10.1155/2014/132301

**Published:** 2014-07-21

**Authors:** Son Minh Nguyen, Minh Khac Nguyen, Mare Saag, Triin Jagomagi

**Affiliations:** ^1^Da Nang University of Medical Technology and Pharmacy, 99 Hung Vuong, Da Nang 59000, Vietnam; ^2^Department of Stomatology, University of Tartu, Raekojapl 6, 51003 Tartu, Estonia

## Abstract

*Objective*. The aim of this study was to evaluate the need for orthodontic treatment among 12-year-old school children and 18-year-olds from Da Nang, Vietnam. *Basic Research Design*. A random representative sample of 200 12-year-old children from primary schools in Da Nang city was gathered. In addition, 200 18-year-old students were randomly selected from among the 4000 students studying at Da Nang University of Medical Technology and Pharmacy, Vietnam. All the subjects were evaluated according to Angle's molar relationship, the presence of malocclusion, and the components of the Index of Orthodontic Treatment Need (Dental Health Component, DHC, and Aesthetic Component, AC). *Results*. The DHC of index of orthodontic treatment need (IOTN) for 12-year-olds was in 60% of cases *no or little*, in 21% of cases *moderate*, and in 19% of cases *definitive*, while the prevalence of moderate and definitive need for treatment among the 18-year-olds was 24% and 30.5%, respectively. The prevalence of class III malocclusion, contact point displacement, and crossbite was higher in 18-year-olds than among the 12-year-olds, while the prevalence of increased overjet and increased overbite had decreased in 18-year-olds compared to the group of 12-year-olds. *Conclusions*. There is a strong need for orthodontic treatment in Vietnam's population. The need for orthodontic treatment was determined by contact point displacement, crossbite, increased overjet, and increased overbite.

## 1. Introduction

The need for orthodontic treatment is influenced by a number of factors including cultural, parental, peer, and self-perception of dental beauty. The assessment of malocclusion and treatment need for public health purposes is crucial for planning orthodontic care service in terms of planning necessary human and financial resources, and also for monitoring the oral health programs offered. Such data are not available for the population of Vietnam.

Some indices have been developed to objectify treatment need. These include the Dental Aesthetic Index (DAI) [1], The California Modification of the Handicapping Labiolingual Deviation Index, the HLD (CalMod) index [2]. The aforementioned indices are useful to determine resource allocation in particular populations or communities, to select those patients who can be treated in a particular dental care system, and to establish priorities when resources are limited.

Brook and Shaw [3] introduced the Index of Orthodontic Treatment Need (IOTN). The IOTN classifies malocclusions according to the presence of particular occlusal features considered important for dental health and aesthetics in order to identify individuals who would benefit most from orthodontic treatment. The IOTN has two separate components: a clinical component referred to as the Dental Health Component (DHC) and Aesthetic Component (AC); DHC with five severity levels and an AC with ten severity levels. There was no attempt to combine these two components and both are recorded separately. The IOTN has been used for this purpose in many epidemiological studies.

Assessment of orthodontic treatment need and demand helps in planning orthodontic services. In Malaysia [4], 47.9% of school children had a definite need for orthodontic treatment, and the percentage was 37% among Hong Kong Chinese [5]. Livas and Delli [6] systematically reviewed the literature concerning orthodontic treatment need and established that the prevalence of definite need of DHC-IOTN is often greater than 11%, and extends up to 70%, although results are affected by gender, age, and region.

Vietnam is the easternmost country on the Indochina Peninsula in Southeast Asia; Da Nang is the biggest city on the South Central Coast of Vietnam and is the commercial and educational center of Central Vietnam. Little published information is available about the oral health status of the population of Vietnam. The present study aimed to evaluate the need for orthodontic treatment among 12-year-old Vietnamese school children and 18-year-old young adults. The findings may be of value for providing orthodontic and human resource training, treatment facilities, and resource planning.

## 2. Material and Methods

The investigation targeted 200 12-year-old children, who were randomly selected from five primary schools and 200 18-year-old students, randomly selected from among the 4000 students studying at Da Nang University of Medical Technology and Pharmacy. The subjects had no history of orthodontic treatment. A minimum sample size consisting of 384 individuals was calculated on the premise of a prevalence of 50% for unknown orthodontic treatment need in Da Nang, with a standard error of 5% and a 95% confidence interval. A round study sample was set at 400 subjects. The study was performed according to the guidelines of the Research Ethics Committee of the Da Nang University of Medical Technology and Pharmacy.

School children were examined in the dental clinics of their primary schools and the students were examined in the dental clinic of Da Nang University of Medical Technology and Pharmacy without external interference. The examination lasted approximately 15 minutes per participant, following the World Health Organization (1997) guidelines. The assessment of dental occlusion was carried out using latex gloves, dental mirrors, and a CPI probe. No radiographs, study casts, or previous written records were used. Two general dentists evaluated Angle's molar relationship, the presence of malocclusion, and components of the IOTN. Intrarater reliability was assessed during the training session; all the examination of DHC and AC score were assessed two times in a group of 20 subjects by the same dentist (the first author) who was supervised by a certified orthodontist; the time interval between examinations was 2 days and Kappa index was utilized and assessed to be 0.86 for DHC and 0.91 for AC score.

Orthodontic variables including Angle's molar relationship and presence of malocclusion (increased overjet, increased overbite, crossbite, and contact point displacement) were evaluated according the World Health Organization (1997) guidelines. The need for orthodontic treatment was assessed by means of the DHC of the IOTN [3]. The DHC of the IOTN has five grades: grades 4 and 5 represent high priority for treatment, grade 3 is a borderline need, and grades 1 and 2 are little or no need for treatment. The AC consists of ten scaled color photographs showing different levels of dental attractiveness. Grade 1 represents the most attractive tooth arrangement and grade 10 the least attractive arrangement of teeth. When the AC was being recorded, the dental attractiveness of the anterior teeth was graded by an examiner after the subjects had closed their teeth in central occlusion and retracted their lips.

### 2.1. Analysis

The data were analyzed using version 18.0 SPSS software. Descriptive statistics and the chi square correlations between the orthodontic variables were calculated. Student's *t-*test was used to compare the prevalence of orthodontic treatment need among the two groups. A significance level of 5% was considered relevant.

## 3. Results

The study encompasses a sample of 200 12-year-old children and 200 students of 18 years of age, with an overall distribution of 54.5% female and 45.5% male.

The prevalence of moderate or definite orthodontic treatment need of Dental Health Component scale was recorded in 47.5% of the sample and Aesthetic Component in 22.8% of cases (Table 1). The prevalence of a definite need according to the AC scale is higher in females than in males (*P* < 0.05). There was no statistically significant difference for orthodontic treatment need according to DHC between the age groups (*P* > 0.05).

The prevalence of malocclusion and occlusal traits in the total sample was also recorded. The prevalence of class I, II, and III malocclusion was measured at 67%, 17.5%, and 15.5%, respectively. An increased overjet (>3.5 mm) was present in 36.3% of subjects while 26.3% had increased overbite (>3.5 mm). Contact point displacement was found in 54% of the subjects examined and 22.3% had crossbite. A significant difference was observed between malocclusion, increased overjet, crossbite, displacement, and increased overbite between the age groups.

The statistically significant differences of each occlusal trait, involving orthodontic treatment need of DHC, in the total sample are presented in Table 2 (*P* < 0.05). With subjects showing overjet greater than 3.5 mm, the treatment need was 52.5%, while 78.2% subjects with contact point displacement of more than 1 mm were assessed to have an objective need for orthodontic treatment; crossbite and increased overbite were present in 82.4% and 62.9%, respectively, of cases.

**Figure 1:**
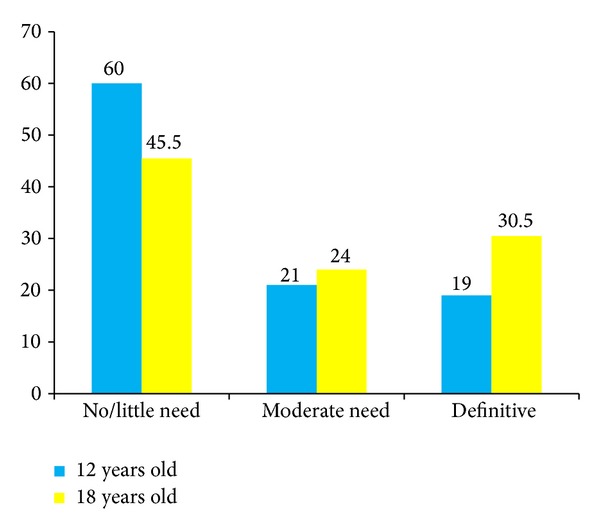
Frequency of grades DHC by age groups.

Among the 12-year-olds, 21% had a moderate (grade 3) and 19% had a definite (grades 4-5) need for orthodontic treatment (Figure 1). The prevalence of moderate and definite treatment need in the sample of 12-year-olds was lower than in the sample of 18-year-olds.

## 4. Discussion

IOTN was used to evaluate the need for orthodontic treatment and to evaluate the severity of malocclusion. The investigation was performed on subjects from groups of 12- and 18-year-olds in Da Nang, Vietnam. According to the DHC of IOTN results, 52.8% of subjects were in little need of treatment (grades 1-2), 22.5% had a moderate need for treatment (grade 3), and 24.7% had a definitive need for treatment (grades 4-5). A statistically significant difference was identified between the age groups. Several studies in Asia have now reported on the prevalence of the need for treatment among the 12- to 18-year-old age group. For example, a previous study indicated that 70% of 12-year-old Hong Kong Chinese school children had an objective need for treatment (grades 3–5) [5]; a study conducted in Shiraz, Iran, reported a prevalence of 51.7% [7]; an examination of 12-year-olds in Malaysia recorded 47.9% [4], while the present study suggests that the need for orthodontic treatment is 47.2%. This percentage score is higher than that recorded for the Nigerian population [8] and in Valencia, Spain [9].

With regard to the distribution of AC-IOTN, a definitive need (grades 8–10) was identified among 6.5% of the subjects, a moderate need (grades 5–7) among 16.3%, and 77.2% of the sample showed little need for treatment (grades 1–4). The need for orthodontic treatment among the Vietnamese sample did vary significantly by gender. Male subjects can have greater maxillary dimension widths than female subjects, and the crowding of anterior teeth is more likely among girls than boys. Similar results were obtained in a study conducted on the school population of Zahedan, Iran [10], and among Brazilian school children [11]. However, the category of definitive need according to AC recorded in the present study was higher than in the study of Ngom et al. [12] and lower than that recorded by Abdullah and Rock [4].

There was a low agreement between DHC and AC in this study because DHC in the present study was evaluated by occlusal anomalies while evaluating AC, and the use of photographs of dentition limits overjet and lip-incisor evaluations [13]. Similar results had been found previously [14, 15]. However, AC assesses the aesthetic aspects of malocclusion and plays a role in the sociopsychological impact of malocclusions for young people [16]. Class III malocclusion is a challenging orthodontic problem that is common in the Asian population [17, 18]. According to Angle's classification in this study, the prevalence of class I, II, and III malocclusion was 67%, 17.5%, and 15.5%, respectively. We found an increasing prevalence of class III malocclusion between age groups from 10.5% among the 12-year-olds to 20.5% for the 18-year-olds. The results also showed that 66.1% of subjects had a treatment need according to DHC in the group of class III malocclusion (*P* < 0.001). The result revealed a statistically significant difference between age groups for class III malocclusion, increased overjet, contact point displacement, increased overbite, and crossbite (*P* < 0.05). The prevalence of class III malocclusion, contact point displacement, and crossbite was greater among 18-year-olds than among 12-year-olds, while the prevalence of increased overjet and increased overbite was lower for 12-year-olds than among the 18-year-old group. This finding is in line with that of De Baets et al. [11]. Significant changes in total mandibular length continue into young adulthood with increases between late maturation stages [15, 19]. In this study, for the 18-year-old group, increasing mandibular length leads to the lower first molars moving forward and consequently to an increase in class III malocclusion, compared to the prevalence among 12-year-olds. In addition, all teeth were present in the dental arch after 12 years of age, which can cause a lack of space for teeth and increased crowding and also lead to teeth moving in the frontal direction, causing crossbite.

## 5. Conclusions

According to the DHC of the IOTN, 47.2% of the 12-year-old school children and 18-year-old young adult population of Da Nang are in need of orthodontic treatment, as indicated by the proportions of contact point displacement, crossbite, increased overjet, and increased overbite among that group.

## Figures and Tables

**Table 1:** Distribution of molar relationship, AC, DHC, and malocclusion in relation to gender and age group.

Variables	*n* (%)	Gender		*P* value	Age groups		*P* value
		Female	Male		12	18	
		*n* (%)	*n* (%)		*n* (%)	*n* (%)	
Angle relationship							
Class I	268 (67)	158 (59)	110 (41)	0.79	144 (72)	124 (62)	0.03∗
Class II	70 (17.5)	41 (58.6)	29 (41.4)	0.99	35 (17.5)	35 (17.5)	1.00
Class III	62 (15.5)	35 (56.5)	27 (43.5)	0.72	21 (10.5)	41 (20.5)	0.006∗
Aesthetic Component (AC)							
No/little need	309 (77.2)	184 (59.5)	125 (40.5)	0.43	156 (78)	153 (76)	0.72
Moderate need	65 (16.3)	30 (46.2)	35 (53.8)	0.02∗	35 (17.5)	30 (15)	0.49
Definite need	26 (6.5)	20 (76.9)	6 (23.1)	0.04∗	9 (4.5)	17 (8.5)	0.10
Dental Health Component (DHC)							
No/little need	211 (52.8)	115 (54.5)	96 (45.5)	0.86	120 (60)	91 (45.5)	0.04∗
Moderate need	90 (22.5)	60 (66.7)	30 (33.3)	0.74	42 (21)	48 (24)	0.47
Definite need	99 (24.7)	59 (59.6)	40 (40.4)	0.79	38 (19)	61 (30.5)	0.08
Increased overjet (>3.5 mm)	145 (36.3)	71 (49)	74 (51)	0.08	93 (46.5)	52 (26)	0.000∗
Contact point displacements (>1 mm)	216 (54)	131 (60.6)	85 (39.4)	0.345	87 (43.5)	129 (65)	0.000∗
Increased overbite (>3.5 mm)	105 (26.3)	55 (52.4)	50 (47.6)	0.138	62 (31)	43 (21.5)	0.03∗
Crossbite	91 (22.8)	57 (62.6)	34 (37.4)	0.362	30 (15)	61 (30.5)	0.000∗

*Significant differences in *t*-test results with 95% confidence interval.

**Table 2:** Prevalence of malocclusions in the total sample according to the level of need for orthodontic treatment.

Malocclusion	*N* (%)	Treatment need-DHC			*P* value
		No/little	Moderate	Definite	
		*N* (%)	*N* (%)	*N* (%)	

Increased overjet (>3.5 mm)	145 (36.3)	69 (47.5)	33 (22.8)	43 (29.7)	0.036∗
Contact point displacements (>1 mm)	216 (54.0)	47 (21.8)	80 (37.0)	89 (41.2)	0.000∗
Increased overbite (>3.5)	105 (26.3)	39 (37.1)	32 (30.5)	34 (32.4)	0.001∗
Crossbite	91 (22.8)	16 (17.6)	31 (34.1)	44 (48.3)	0.000∗

*Significant difference in chi-square test with 95% confidence interval.
